# Senescent Cardiac Fibroblast Is Critical for Cardiac Fibrosis after Myocardial Infarction

**DOI:** 10.1371/journal.pone.0074535

**Published:** 2013-09-11

**Authors:** Fuli Zhu, Yulin Li, Junmeng Zhang, Chunmei Piao, Tingting Liu, Hui-Hua Li, Jie Du

**Affiliations:** 1 Beijing AnZhen Hospital, Capital Medical University, Beijing Institute of Heart, Lung and Blood Vessel Diseases, the Key Laboratory of Remodeling-related Cardiovascular Diseases, Ministry of Education, Beijing, China; 2 Department of Physiology and Pathophysiology, Capital Medical University, Beijing, China; National Centre for Scientific Research, 'Demokritos', Greece

## Abstract

Senescence is a recognized mechanism of cardiovascular diseases; however, its contribution to myocardial fibrosis and rupture after infarction and the underlying mechanisms remain unclear. Here we showed that senescent cardiac fibroblasts markedly accumulated in heart after myocardial infarction. The expression of key senescence regulators, especially p53, was significantly up-regulated in the infarcted heart or hypoxia-treated fibroblasts. Furthermore, knockdown of endogenous p53 by siRNA in fibroblasts markedly reduced hypoxia-induced cell senescence, cytokine expression but increased collagen expression, whereas increased expression of p53 protein by adenovirus infection had opposite effects. Consistent with in vitro results in cardiac fibroblasts, p53 deficiency in vivo significantly decreased the accumulation of senescent fibroblasts, the infiltration of macrophages and matrix metalloproteinases, but enhanced collagen deposition after myocardial infarction. In conclusion, these results suggest that the p53-mediated fibroblast senescence limits cardiac collagen production, and inhibition of p53 activity could represent a novel therapeutic target to increase reparative fibrosis and to prevent heart rupture after myocardial infarction.

## Introduction

Myocardial infarction (MI), one of the leading causes of mortality in aged people, leads to complex structural remodeling. Following MI, infarct healing is immediately initiated, including the infiltration of inflammatory cells, activation of matrix metalloproteinases (MMPs), myofibroblast production of extracellular matrix and scar formation [1,2]. Both clinical and experimental studies have demonstrated aging-associated defects in inflammation, collagen deposition and cardiac repair, which contribute to adverse remodeling including ventricular dilation and hypertrophy [3,4]; however, the molecular mechanisms for the cell senescence of myocardial infarction have not yet been elucidated.

Cellular senescence is a process of growth-arrest that limits the proliferation of mammalian cells [5]. Senescent cells are characterized by several molecular and cytological markers, including a large flattened morphology, up-regulation of senescence-associated β-galactosidase (SA-β-gal) activity and proteins (such as p16, p19, p21 and p53) [6]. Several pathways can induce senescence in various cell types [7]. Among them, p53/p21 pathway has a key role in the induction of cell senescence. Elevated p53 activity can induce senescence in proliferative tumor cells and other cell types [8,9,10], whereas inhibition of the p53 activity in senescent cells can reverse the phenotype [11]. Increased p53 activity also induces cell apoptosis in response to diverse pathological stresses such as ischemia and myocardial infarction [12,13,14]. However, whether p53-mediated cell senescence influences cardiac remodeling after infarction remains unknown.

In the present study, we examined the role of cellular senescence in regulating cardiac fibrosis after myocardial infarction. Our results demonstrated that myocardial infarction or H/R promotes fibroblast senescence and the expression of key senescence regulators, especially p53, which decrease collagen production and the reparative cardiac fibrosis, contributing to cardiac rupture. Changes in p53 levels regulated these effects. Thus, these results suggest that p53-mediated fibroblast senescence inhibits cardiac fibrosis after myocardial infarction.

## Materials and Methods

### Antibodies and Reagents

Senescence-associated β-galactosidase (SA-β-gal) activity assay kit was purchased from Abcam (Cambridge, MA). The antibodies against p53, α-smooth muscle actin (α-SMA), 488-goat anti-mouse, 555-goat anti-rabbit and cy3-donkey anti-goat were from Cell Signaling Technology (Beverly, MA); antibodies against p16, p19, p21, discoidin domain receptor 2 (DDR2), troponin I and Mac-2 were from Santa Cruz Biotechnology (Santa Cruz, CA). Penicillin, streptomycin, fetal bovine serum (FBS) and others were obtained from Invitrogen Life Technologies (Carlsbad, CA) or Sigma (Sigma-Aldrich, Louis, MO).

### Animals and myocardial infarction model

Wild-type (WT) littermates and homozygous p53 knockout mice (p53 KO) on C57/B6 background were obtained from the Jackson Laboratory as described [14]. WT and p53 KO male mice (8- to 12-week-old) were anesthetized with 2% isoflurane inhalation and subjected to operation of myocardial infarction model by ligation of left coronary artery (LCA) as described [15]. The sham group underwent the same surgical procedure except that the LCA was not occluded. Mice were sacrificed at 7^th^ day post-operation and heart tissues were harvested. All animal protocols were approved by the Animal Care and Use Committee of Capital Medical University (20120112) and experiments conformed to the Guide for the Care and Use of Laboratory Animals (National Institutes of Health publication No. 85-23,1996).

### Histology and immunohistochemistry

Heart tissues were fixed in 4% paraformaldehyde, embedded in paraffin and sectioned at 5 µm intervals. Hematoxylin/eosin (H&E), Masson’s trichrome and Sirius Red staining were performed using standard procedures as described [16,17]. The percentage of collagen deposition (blue or red staining as positive area, respectively) to ischemic tissue was analyzed and calculated.

Immunostaining was performed as described previously [18,19]. Heart sections were stained with antibodies against troponin I (1:200), p53 (1:200), α-SMA (1:200), DDR2 (1:200), p16 (1:200) and p21 (1:200) at 4°C overnight. Followed by incubation with Alexa Fluor conjugated secondary antibodies. DAPI was used for counterstaining. An irrelevant isotype mouse, rabbit or goat IgG was used as a negative control. Immunohistochemistry was performed on paraffin heart sections (5 µm) with antibodies against p16 (1:200), p19 (1:200), p21 (1:200), p53 (1:200) and Mac-2 (1:200) at 4°C overnight [18,19]. Biotin-conjugated secondary antibody was added for 30 min at room temperature. For color development, we used 3, 30-diaminobenzidine tetrahydrochloride (DAB) and hematoxylin as a counterstain. Images were viewed and captured using a Nikon Labophot 2 microscope.

Senescence-associated β-galactosidase (SA-β-gal) activity assay in tissues and cells was performed using Cellular Senescence Detection Kit according to manufacture’s protocol. Briefly, cells or frozen heart sections were fixed in 1% formaldehyde for 5 min. After washing with PBS, sections were incubated with 1 mg/ml X-Gal staining solution overnight at 37°C, and then counterstained with Eosin. Blue-stained senescent cells were observed under light microscopy and counted.

The number of staining positive cells were calculated by using NIS-ELEMENTS quantitative automatic program (Nikon, Tokyo, Japan) with the average value of at least 10 random fields from each section in double-blind fashion. The percentage of staining positive cells was determined by the number of cells having positively staining divided by the total counted cells and multiplied by 100.

### Cell culture, siRNA transfection and adenovirus infection

Cardiac fibroblasts (CFs) were isolated and cultured as described [18,19]. Briefly, hearts excised from neonatal WT mice (1-3 day-old) were minced and digested. The supernatant was filtered through a 400-µm nylon mesh filter and then centrifuged at 800 rpm for 5 min at 4°C. Cells were placed into dishes with fibroblast culture media (DMEM, 10% normal bovine serum albumin and 100 U/ml penicillin/streptomycin) and incubated at 37°C with 5% CO_2_ and 95% air. Fibroblasts (passages 6 to 12) were transfected with 100 nmol/L of small interfering RNA (siRNA) using lipofectamine 2000 according to manufacture’s protocol or were infected with recombinant overexpression adenovirus (Ad), including Ad-GFP or Ad-p53 at multiplicity of infection (50–100). The sequences of double strand siRNA targeting p53 were 5'-CCAGAAGAUAUCCUGCCAUTT-3', 3’-AUGGCAGGAUAUCUUCUGGTT-5’ (purchased from Invitrogen, San Diego, CA).

### Simulated hypoxia/reoxygenation protocol

The simulated hypoxia/reoxygenation (H/R) was performed as previously described [20,21]. After 24 hrs of adenovirus infection, the cells were subjected to hypoxia buffer that contained 118 mM NaCl, 24 mM NaHCO_3_, 1.0 mM NaH_2_PO_4_, 1.2 mM MgCl_2_, 2.5 mM CaCl_2_–2H_2_O, 16 mM KCl, 20 mM sodium lactate, 10 mM 2-deoxyglucose (pH 6.2) for 3 h. Reperfusion was accomplished by replacing the hypoxia buffer with normal cell medium under normoxia conditions for 3 days.

### Quantitative real-time PCR

Total RNAs were isolated from cultured fibroblasts or fresh mouse hearts by the Trizol method (Invitrogen). Samples (1-2 µg) were reverse-transcribed to generate first-strand cDNA by use of MMLV Reverse Transcriptase (Promega). Quantitative real-time PCR (qPCR) analysis was performed with iQ5 Real-Time PCR Detection System (Bio-Rad, Hercules, CA) and specific primers for mouse IL-6, IL-11, CXCL1, MMP2, MMP9, collagen I, and collagen III as described previously [19,22,23]. A housekeeping gene β-actin was used as an internal standard.

### Analysis of cell proliferation

For growth curves, cells (1x10^4^ cells per well) were grown in 12-well plates and treated as indicated. Cells were stained with 1% Trypan blue and the numbers of total cells were counted. For BrdU incorporation, cells in 24-well plates (5x10^3^ per well) were treated with H/R for 3 days and controls were left untreated. BrdU was labeled according to manufacturer’s protocol. BrdU positive cells were visualized with secondary antibodies conjugated with 488-goat anti-mouse. Fluorescence microscopy images were taken from 8 random fields in each well using NIKON camera (NIS-ELEMENTS quantitative automatic program).

### Western Blot Analysis

Cells were lyzed with lysis buffer (20mM Tris (pH7.5), 1mM EDTA, 150mM NaCl, 1 mM EGTA, 1 mM β-glycerophosphate, 1% Triton X-100, 2.5 mM sodium pyrophosphate, 1 mM Na _3_VO_4_, 4µg/ml aprotinin, 4 µg/ml leupeptin, 4 µg/ml pepstatin, and 1 mM PMSF). 50 µg protein lysates were separated by 10% SDS-PAGE and then transferred to nitrocellulose membranes, the membranes were incubated with primary antibody against anti-p53 (1:1000), p21 (1:200) and GAPDH (1:5000) at 4°C overnight, then with IR Dye-conjugated secondary antibodies (1:5000) for 1hr. The images were quantified by the use of the Odyssey infrared imaging system (LI-COR Biosciences, Lincoln, NE) as described [17,18,19]

### Measurement of cytokines level

Cardiac fibroblast cells were cultured in 24-well plates infected with adenovirus as mentioned above and treated with simulated H/R for 3 hrs, then replaced in normoxia media for 48 hrs. The culture supernatants were analyzed for the protein levels of cytokines with Bio-Plex assay kit according to manufacturer’s protocols (Bio-Rad).

### Statistical analysis

All data were analyzed using GraphPad software (GraphPad Prism version 4.00 for Windows; GraphPad Software). Results are expressed as mean ± SEM. Differences are analyzed by t test or ANOVA, P < 0.05 was considered statistically significant.

## Results

### Senescent fibroblasts accumulate in the infarcted heart in vivo

**Figure 1:**
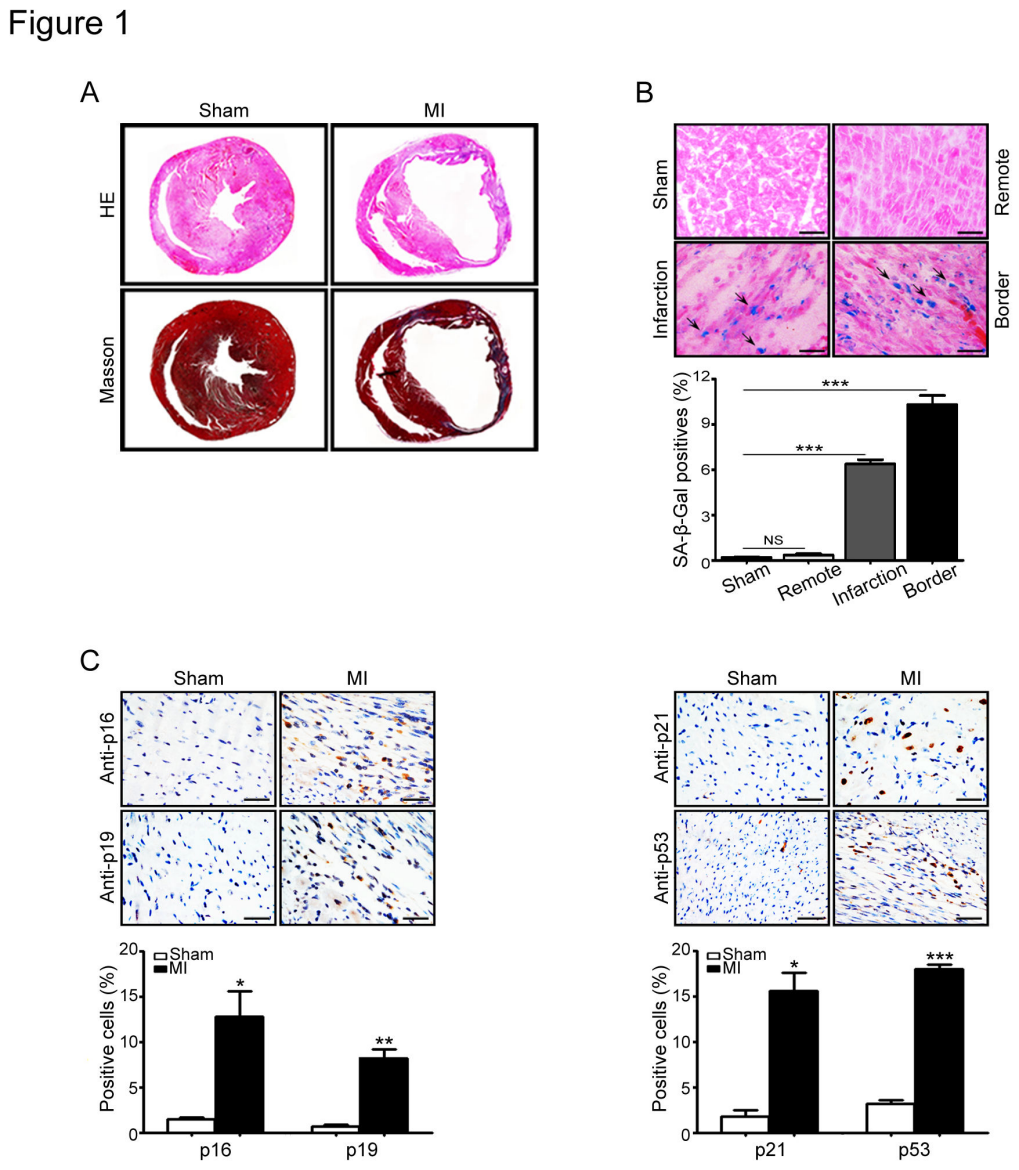
Senescent cells accumulated in heart after infarction. (A) Myocardial infarction (MI) was induced by ligation of left coronary artery (LAC) in mice. After 1 week of MI, the heart sections were evaluated by hematoxylin/eosin (H&E) and Masson staining. (B) Senescent cells were detected by SA-β-Gal staining in the heart (left). Bar graph shows the percentage of SA-β-Gal positive cells in the heart sections (right). (C) A panel of senescence markers, including p16, p19, p21 and p53, was immunostained with specific antibodies (left). Scale bars: 50 µm. The percentage of positive cells was measured by selecting 6 random fields. Bar graphs show the percentage of p16-, p19-, p21- and p53-positive cells in heart sections (right). Data expressed as mean±SEM (n=5 per group). *P<0.05, **P<0.01, ***P<0.001 vs. sham.

To investigate the role of cellular senescence in cardiac fibrosis, myocardial infarction was induced by ligation of left coronary artery (LAC) in male mice and was evaluated by hematoxylin/eosin (H&E) and Masson staining. As shown in Figure 1A, infarction size and fibrotic area were significantly increased in the infarcted heart compared with that in sham control. To identify senescent cells in vivo, heart sections from infracted or sham mice were stained with a panel of senescence-associated markers, including SA-β-gal activity and proteins (p16, p19, p21 and p53), which are associated with cellular senescence process [24,25]. As shown in Figure 1B and C, the number of cell staining positive for SA-β-gal and senescence-associated proteins were all significantly increased in the infarct-border regions of heart compared with shamed heart. Notably, less SA-β-gal positive cells were observed in remote area of the heart where didn’t experience ischemia process or in sham heart. These results indicate that senescent cells accumulate in the heart after infarction.

**Figure 2:**
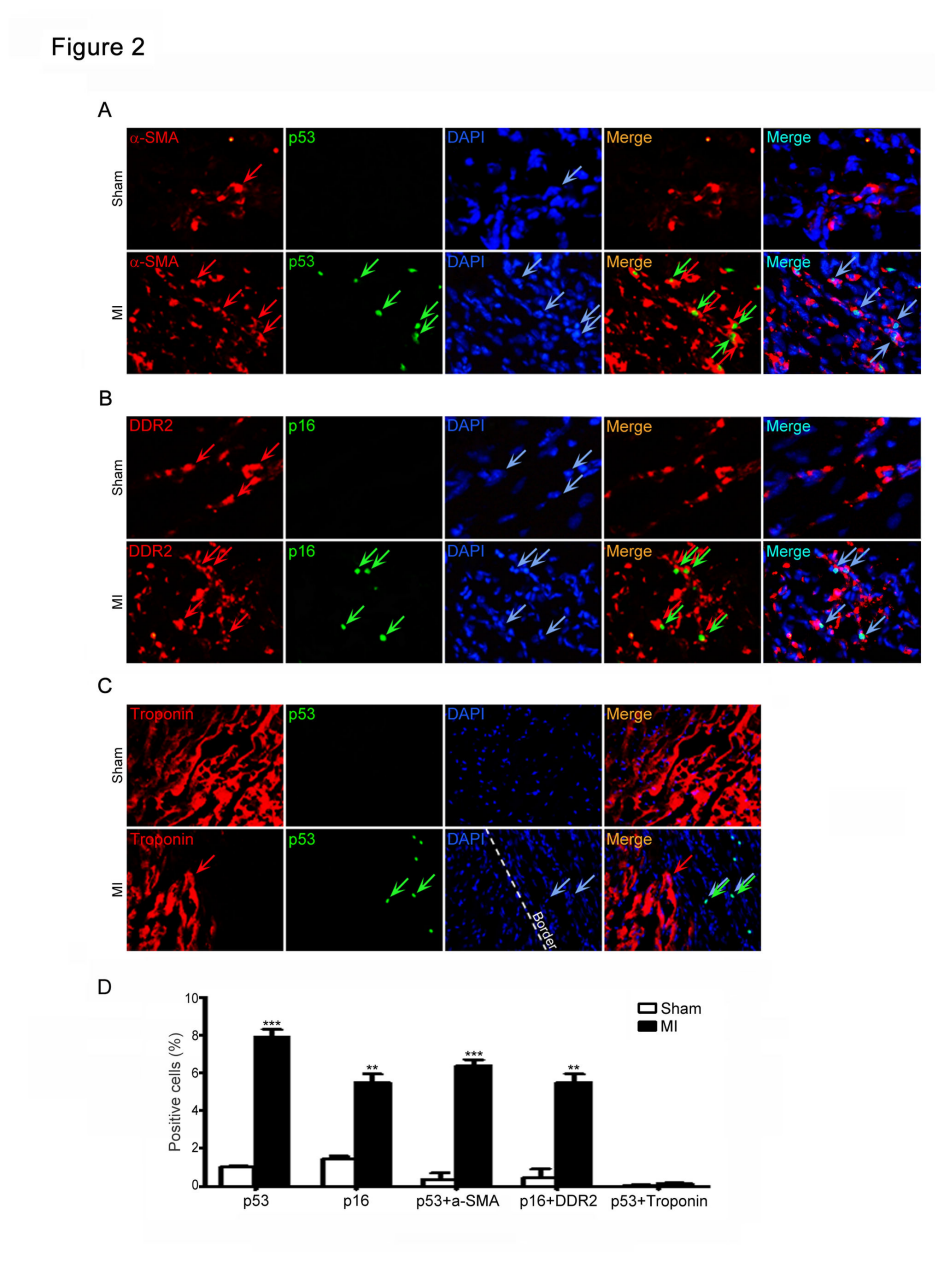
Senescent cells derive from cardiac myofibroblasts. (A, B) Heart tissues at day 7 after infarction were double-stained using anti-p53 or p16 (markers for senescence, green) and anti-a-SMA and DDR2 (markers for myofibroblasts) antibodies, and counterstained with DAPI (blue), and then examined by a fluorescence microscopy. (C) The heart sections were immunostained using the combination of anti-p53 (green) and anti-Troponin (a marker for cardiomyocytes, red) antibodies and counterstained with DAPI (blue), and examined by a fluorescence microscopy. Scale bars: 50 µm. (D) Bar graph shows the percentage of staining positive cells.

Fibroblasts are known to be the most abundant cells in the heart [26,27], the location of senescent cells in fibrotic areas of the heart (both the border and infarcted regions) (Figure 1B and C) suggested that senescent cells may be derived from fibroblasts, which initially proliferate and produce extracellular matrix in fibrosis formation following heart injury [26,27]. Indeed, in the infarcted heart sections, the cells that were stained positive for the senescence-associated markers p53 or p16 were also positive for the myofibroblast markers α-SMA or DDR2, respectively (Figure 2A and B), but not for cardiomyocyte marker troponin I (Figure 2C). Moreover, the percentage of α-SMA- or DDR2-positive myofibroblasts was higher in the infarcted heart than that in sham control (Figure 2A and B). Collectively, these results suggest that myocardial ischemia triggers fibroblast senescence in the heart.

### Hypoxia/reoxygenation induces cardiac fibroblast senescence

**Figure 3:**
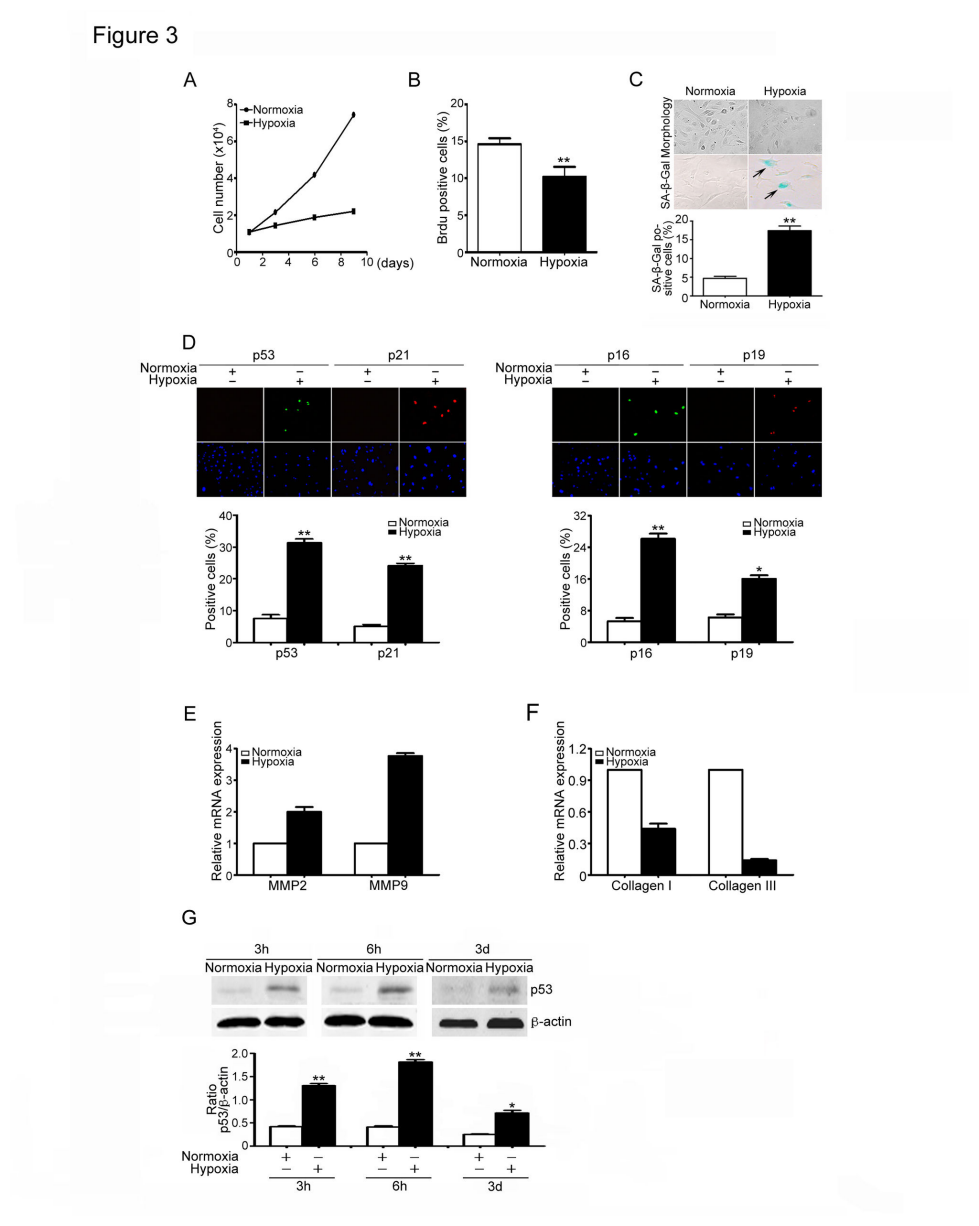
Hypoxia/reoxygenation induces senescence in cardiac fibroblasts. (A) Neonatal cardiac fibroblasts from wild-type (WT) mice were treated with hypoxia/reoxygenation (H/R) for 0-10 days. The growth of viable cells was measured by using Trypan blue staining. Bar graphs show the number of viable cells at day 0-10 of H/R treatment. (B) Cells were culture as in A for 3 days. Cell proliferation was measured by using BrdU incorporation assay. Bar graphs show the percentage of BrdU positive cells. (C) Morphology and SA-b-Gal staining of fibroblasts treated with hypoxia were viewed and performed (left). Bar graph shows the percentage of SA-β-Gal positive cells (right). (D) Cells treated with hypoxia were subjected to immunostaining using anti-p16, p19, p21 and p53 (markers of senescence). DAPI was used for counterstaining (left). Bar graphs show the percentage of senescence marker positive cells (right). (E, F) qPCR analysis was used to quantify the mRNA expression of MMP2, MMP9, collagen I and collagen III in fibroblasts treated with hypoxia. Bar graphs show the relative mRNA levels in hypoxia-treated cells compared with normoxia group. (G) p53 protein levels were detected by Western Blot analysis in fibroblasts treated with hypoxia for 3 h, 6 h and 3 d. Bar graphs show the quantitative analysis of p53 protein (right). Scale bars: 50 µm. Data expressed as mean±SEM (n=3). *P<0.05, **P<0.01, ***P<0.001 vs. normoxia.

Hypoxia has been reported to induce premature senescence in neonatal rat cardiomyocytes, fibroblast and bone marrow hematopoietic cells [28,29,30]. Ischemia or H/R promotes differentiation of fibroblasts to myofibroblasts in pulmonary arteries [31,32], we therefore examined whether H/R promotes cardiac fibroblast senescence in vitro. Cardiac fibroblasts were cultured at H/R for 0-10 days. The viability, proliferation and senescence of fibroblasts were evaluated by Trypan blue staining (cell viability). BrdU incorporation and expression of senescence-associated markers. H/R treatment significantly reduced the number of viable cells in a time-dependent manner (Figure 3A) and inhibited proliferation of cardiac fibroblast at day 3 of treatment as compared with normoxia-treated cells (Figure 3B). Moreover, H/R-treated cardiac fibroblasts exhibited enlarged and flattened cell morphology, which are the typical characteristics of cellular senescence (Figure 3C). Furthermore, the activity of SA-β-gal and expression of senescence-associated markers including p16, p19, p21 and p53 were all markedly higher in the hypoxia-treated cells than that in normoxia control (Figure 3C and D). In addition, the levels of MMP2 and MMP9 mRNA expression were significantly up-regulated, but the mRNA expression levels of collagen I and III were markedly decreased in hypoxia-treated cells than that in normal cells (Figure 3E and F). Finally, hypoxia markedly up-regulated the expression of p53 protein in a time-dependent manner (Figure 3G). Together, these results suggest that Hypoxia induces cardiac fibroblast senescence and p53 may play a critical role in this process.

### Effect of p53 on hypoxia-induced fibroblast senescence

**Figure 4:**
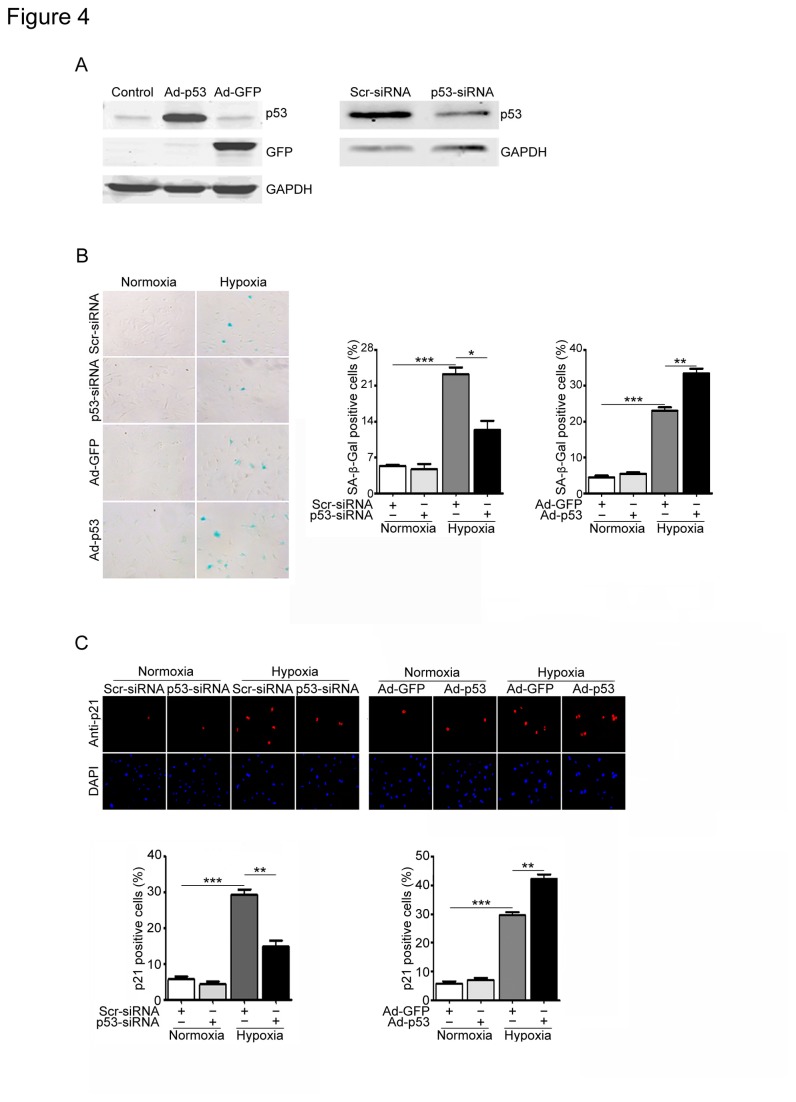
Effects of p53 on cell senescence and p21 expression in cardiac fibroblasts induced by hypoxia/reoxygenation. (A) Cardiac fibroblasts were infected with scrambled siRNA (Scr-siRNA, 100 nmol/L), p53-siRNA (100 nmol/L), or adenovirus GFP control (Ad-GFP, MOI=50) or p53 (Ad-p53, MOI=50) for 24 h and then exposed to hypoxia/reoxygenation (H/R) for 3 days. The infection efficiency was detected by Western blot analysis using anti-p53 antibody. (B) Senescent cells were detected by SA-β-Gal staining (left). Bar graph shows the percentage of SA-β-Gal-positive cells (middle and right). (C) Senescent cells were subjected to immunostaining using anti-p21 antibody (top). DAPI was used for counterstaining (middle). Bar graph shows the percentage of p21-positive cells (bottom). Scale bars: 50 µm. Data expressed as mean±SEM (n=3). ***P<0.001 vs. normoxia; *P<0.05, **P<0.01 vs. Scr-siRNA+hypoxia or Ad-GFP+hypoxia.

Since the p53/p21 pathway plays a critical role in the senescence in several cell types [7], and ischemia or hypoxia markedly induced the expression of p53 and p21 in the infracted heart and in vitro fibroblasts (Figure 1 through 3). We then examined effect of p53 on proliferative capacity and senescence of cardiac fibroblasts. Western blot analysis revealed that the endogenous p53 expression was markedly reduced by infection of p53 siRNA (p53-siRNA) but not scrambled siRNA (Scr-siRNA) (Figure 4A, left). After hypoxia treatment, the number of SA-β-gal-positive cells was significantly decreased in the p53-siRNA-infected cells compared with that in scrambled siRNA (Figure 4B, left and middle). Moreover, immunostaining demonstrated that knockdown of p53 markedly reduced p21-positive cells, a main downstream target of p53, compared with scrambled siRNA after hypoxia stimulation (Figure 4C, left). To further confirm the effect of p53 on cardiac fibroblast, the fibroblasts were infected with adenovirus GFP-vector (Ad-GFP) or p53 (Ad-p53). Infection of Ad-p53 increased 3.7-fold compared with Ad-GFP control after 24 hrs (Figure 4A, right). Furthermore, infection of fibroblasts with Ad-p53 markedly increased the number of SA-β-gal- and p21-positive cells compared with Ad-GFP infection after hypoxia (Figure 4B, left and right and 4C, right). There was no difference in SA-β-gal activity or p21 expression between two groups under normoxia (Figure 4B and C). Thus, inhibition of p53 attenuates fibroblast senescence induced by hypoxia.

### Effect of p53 on the expression of inflammatory factors in fibroblasts in response to hypoxia

**Figure 5:**
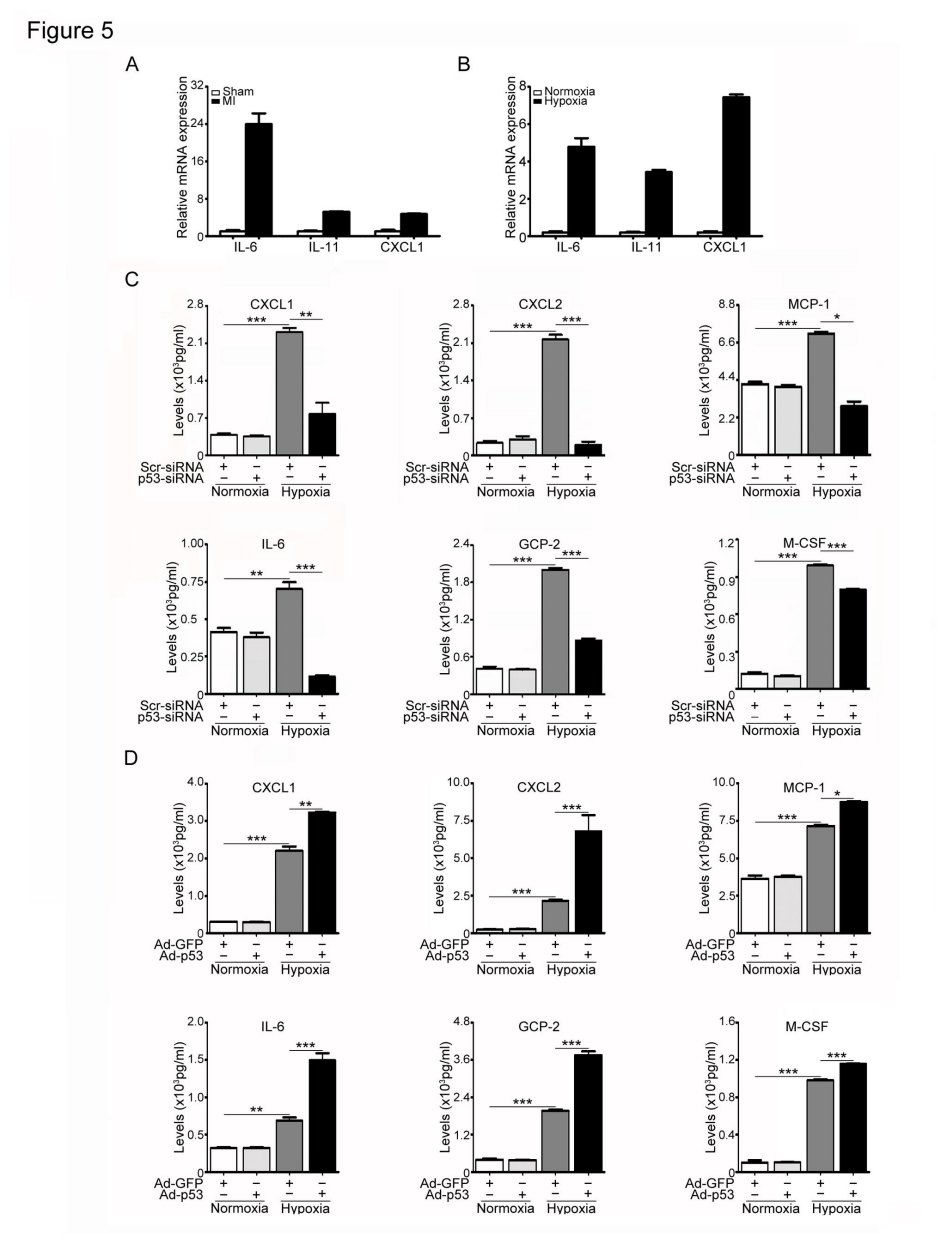
Effect of p53 on the expression of inflammatory factors. (A) Myocardial infarction (MI) was induced by ligation of left coronary artery (LAC) in mice for 1 week. The mRNA expression of IL-6, IL-8 and CXCL1 was examined by qPCR analysis in the heart. Bar graphs show the relative mRNA levels in p53 KO mice compared with WT mice. Data expressed as mean±SEM (n=5 per group). (B) Cardiac fibroblasts were treated as in Figure 4A. The protein levels of IL-6, IL-11, CXCL1, MCP-1, GCP-2, M-CSF and CXCL2 were measured by Bio-Plex assay kit. (D) Cardiac fibroblasts were treated as in Figure 5A. The protein levels of IL-6, IL-11, CXCL1, MCP-1, GCP-2, M-CSF and CXCL2 were measured as in B. Data expressed as mean±SEM (n=3). ***P<0.001 vs. normoxia; *P<0.05, **P<0.01, ***P<0.001 vs. Scr-siRNA+hypoxia or Ad-GFP+hypoxia.

Because cellular senescence in fibroblasts and other cells is accompanied by a marked increase in the production of a wide range of growth factors, chemokines and cytokines (termed senescence associated secretory phenotype) [24]. We then measured several cytokine levels with qPCR analysis. The mRNA levels of IL-6, IL-11 or CXCL1 were significantly increased in the infarcted heart or in hypoxia-treated cells compared with control groups (Figure 5A and B). Moreover, Bio-Plex multiplex assay revealed that p53 knockdown by siRNA significantly reduced the protein levels of CXCL1, CXCL2, MCP-1, IL-6, GCP-2 and M-CSF in fibroblasts after hypoxia exposure compared with scrambled siRNA control (Figure 5C). On the other hand, increased expression of p53 had the opposite effects (Figure 5D). These data indicate that inhibition of p53 decreases inflammatory cytokine expression in senescent fibroblasts in response to hypoxia.

### Knockout of p53 attenuates fibroblast senescence and inflammation but enhances collagen deposition in mouse heart after infarction

**Figure 6:**
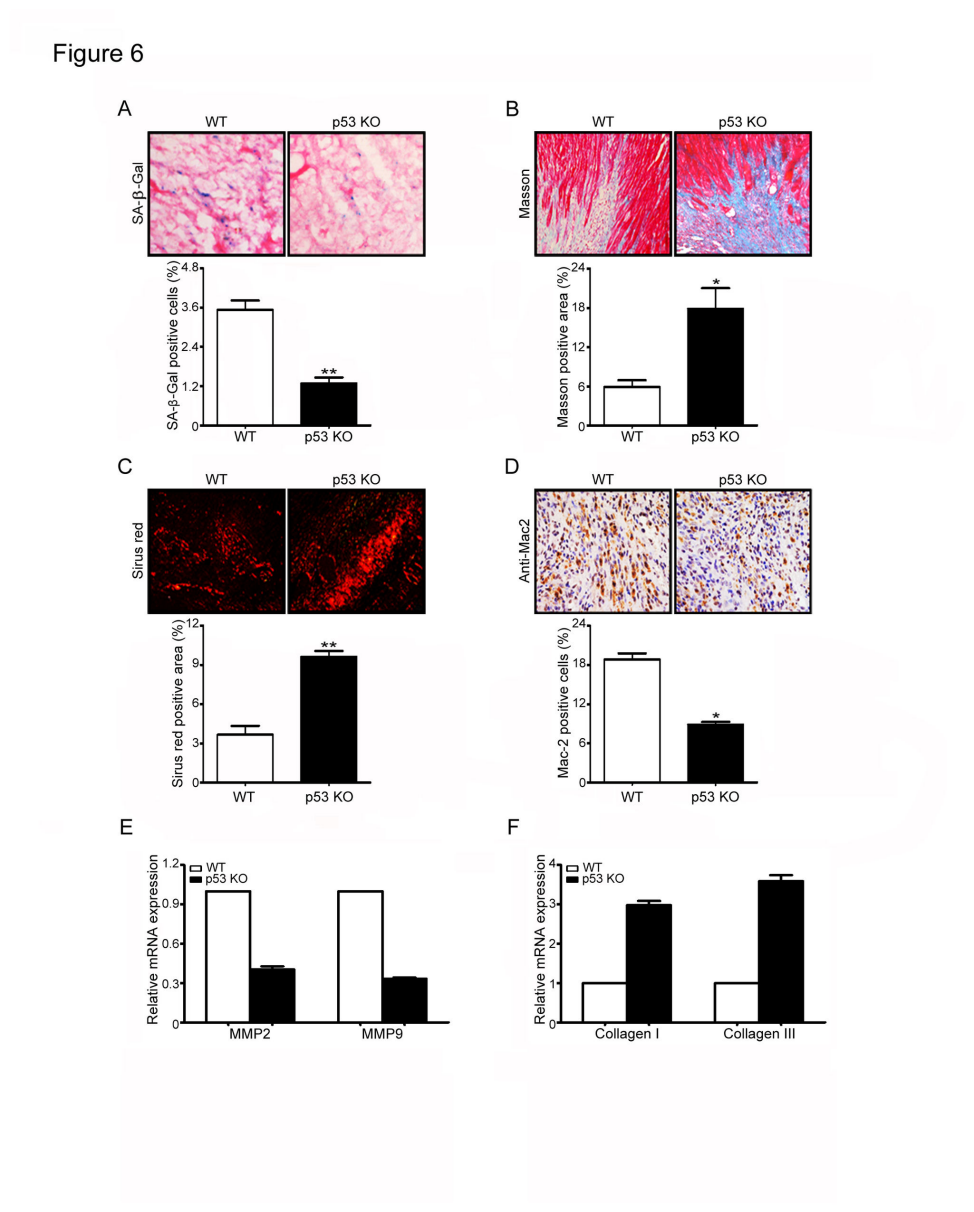
Deficiency of p53 inhibits fibroblast senescence but enhances cardiac fibrosis after infarction. (A) Wild-type (WT) and p53 knockout (p53 KO) mice underwent left coronary artery ligation for 7 days. Heart sections were stained by SA-β-Gal kit (left). Bar graph shows the percentage of SA-β-Gal-positive cells in the heart (right). Heart sections were examined using Masson’s trichrome (B) or Sirius Red staining (C) (left). Bar graphs show the areas of collagen deposition in the heart (right). (D) Heart sections were stained by immunohistochemistry with anti-Mac-2 antibody (left). Bar graph shows the percentage of Mac-2 positive cells in the heart. Scale bars: 50 µm. (E, F) qPCR analysis was used to quantify the mRNA expression of MMP2, MMP9, collagen I and collagen III in the heart tissue. Bar graphs show the relative mRNA levels in p53 KO mice compared with WT mice. Data expressed as mean ± SEM (n=5 per group). *P<0.05, **P<0.01 vs. WT+MI.

Because our above results demonstrate that p53 contributes to cardiac fibroblast senescence in the heart and in cultured fibroblasts (Figure 4). To evaluate the impact of p53-mediated senescence on cardiac fibrosis, we compared the histopathology of heart obtained from WT and p53-deficient mice (p53 KO) subjected to left coronary artery ligation. After 7 days, p53 KO mice exhibited a significantly decrease in the number of SA-β-gal-positive fibroblasts (Figure 6A), an increase in the fibrotic areas (Figure 6B and C) and the expression of collagen I and collagen III (Figure 6F), but a decrease in the expression of MMP 2 and MMP 9 compared with WT mice (Figure 6E), and also displayed an decrease in the number of Mac-2-positive macrophages (Figure 6D). There was no significant difference in cell senescence, inflammation and collagen deposition between two groups after sham operation (Data not shown). These results suggest that p53-dependent senescence limits cardiac fibrosis after infarction.

## Discussion

In this study, we provide the evidence of p53 involvement in fibroblast senescence and myocardial fibrosis after infarction. We demonstrated that senescent fibroblasts accumulated in infracted hearts of mice. Both p53 and p21 were significantly up-regulated in infracted heart and hypoxia-treated fibroblasts. Knockout of p53 markedly attenuates hypoxia-induced fibroblast senescence. Conversely, increased expression of p53 had the opposite effects. Moreover, p53-deficient mice showed a decrease in accumulation of senescent fibroblasts and inflammation, and an increase in fibrosis in the infarcted heart. Thus, p53-mediated fibroblast senescence limits the reparative cardiac fibrosis contributing to cardiac rupture after myocardial infarction (MI).

Myocardial infarction leads to cardiac remodeling and heart failure. During the process, myofibroblasts are key cells that replace the damaged and lost cardiomyocytes, and contribute cardiac repair [1,2]. Cellular senescence was first identified as a process that inhibits the proliferation of cultured human cells [33]. Diverse stimuli including oxidative stress, DNA damage, mitogenic signals and other stresses can cause cells to permanently arrest and senesce. Most of these senescence inducers result in the acquisition of multiple senescence markers, including cell cycle arrest, apoptosis resistance, changes in expression of senescence-associated proteins, SA-β-gal activity and the senescence secretory phenotype [7]. It is known that senescent cells accumulate with age in cardiovascular diseases and promote inflammation and cardiac fibrosis [3,4,7]. However, myocardial injury or H/R induces premature (or replicative) senescence in cardiomyocytes, fibroblasts and bone marrow hematopoietic cells, which are fuctionally different with aged cells, such as difference in production of inflammation [28,29,30]. It must be pointed out that hypoxia has different action on senescence in different types of cells. For example, evidences indicate that hypoxia suppresses cellular senescence of human fibrosarcoma cells (HT-p21-9 cells), fibroblast WI-38t cells, mesenchymal stem cells, and other cells [34,35,36]. However, hypoxia can also induce premature senescence in neonatal SD rat cardiomyocytes, cardiac fibroblasts, and Fanconi anemia bone marrow hematopoietic cells, indicating that hypoxia-induced senescence is cell-specific physiological responses [28,29,32]. Furthermore, exposure of cardiac fibroblast to hypoxia stimulates myofibroblast differentiation and reduces cell proliferation [32]. Consistent with these data, the present study demonstrated that myocardial infarction induced accumulation of senescent fibroblasts characterized by the increase in SA-β-gal activity and the expression of p53 and other proteins (Figure 1B and C). These findings were further confirmed in hypoxia-treated cardiac fibroblasts (Figure 3). Collectively, these results suggest that ischemia or hypoxia can induce fibroblast senescence and the expression of p53 in the heart and in cultured fibroblasts.

Senescence response depends on two pathways that are governed by the tumor suppressor proteins p53 and pRb. Both proteins are critical transcriptional regulators that are responsible for cell cycle, DNA damage, and cell death, which involves a number of upstream regulators and downstream effectors [7]. It is generally considered that senescence occurs via the p53 pathway in response to DNA damage and telomere dysfunction, whereas the p16/pRb pathway mediates senescence caused by oncogenic stimuli, chromatin disruption, and other cellular stresses [7]. p53 is a crucial mediator of cellular senescence [10]. The cyclin-dependent inhibitor p21 is the main downstream effector of p53 signaling during senescence. It is known that replicative senescence in human fibroblasts is dependent on the function of p53 and correlates with activation of p53-dependent transcription [37]. We found that p53 overexpression significantly increases senescence markers only under hypoxic conditions (Figure 5). We interpreted that under normal condition, although overexpression of p53 increases its protein level, the p53 activity is lower. In contrast, hypoxia not only increases p53 protein level with the duration of the hypoxia but also promotes the posttranslational modifications of p53 thereby leading to increased p53 activity [38]. Furthermore, the induction of p21 by hypoxia has been described in a p53-dependent and independent manner [38]. Although several studies have demonstrated the critical role of p53 in regulating cardiac remodeling and function after MI [12,13,14], the role of p53 in cardiac fibroblast senescence in response to ischemia or hypoxia remains unknown. The present results demonstrated that ischemia or hypoxia significantly induced p53 expression in the heart and in cultured cardiac fibroblasts. Knockdown of p53 inhibited fibroblast senescence, reduced the expression of p21 and inflammatory cytokines, and increased collagen production in hypoxia-treated cardiac fibroblasts or in the infracted heart (Figures 4 and 6). In contrast, increased expression of 53 reversed these effects (Figure 4). Overexpression of p53 by Ad-p53 infection induced p21 expression but no statistically significant difference was observed under normoxia (Figure 4C). Thus, these data suggest that p53-mediated signaling in fibroblasts may contribute to fibroblast senescence, leading to suppression of collagen production and cardiac fibrosis after myocardial infarction.

A number of studies support the idea that in addition to growth arrest, the change of senescence secretory phenotype is another essential characteristic of senescence [25]. Recent studies in fibroblasts have demonstrated that senescent cells can secrete numerous of factors, including a variety of growth factors, chemokines and cytokines that are known to stimulate inflammation [25]. Several pathways have been reported to regulate the senescence secretory phenotype, such as DNA damage response (DDR) signaling and p53 signaling [25]. A recent study suggests that p38 MAPK activation also induces the secretion of most senescence secretory phenotype associated factors by increasing NF-κB transcriptional activity, and p53 restrained p38 MAPK activation [39]. Several studies demonstrate that p53 inhibits inflammation and cytokine expression such as IL-1, IL-6, IL-12 and IL-8 in macrophages or other non-senescent cells [40,41,42]. However, there are also reports indicating that p53 can directly activate the expression of inflammatory factors such as ICAM-1 in a NF-κB-independent manner in senescent human cells in atherosclerotic lesions and other cell types [43,44]. These results suggest that p53 regulate cytokine expression in cell type-specific fashion. Here, we demonstrated that p53 was a critical senescence regulatory pathway in cardiac fibroblasts in response to ischemia and hypoxia stimuli. Depletion of p53 markedly reduced the secretion of several senescence factors, including IL-1 CXCL1, CXCL2, MCP-1, IL-6, GCP-2 and M-CSF in cardiac fibroblasts and in the infarcted heart, whereas increased expression of p53 enhanced senescence protein expression (Figure 5). Thus, our identification of the p53 pathway as a necessary regulator of the senescence provides new insights into how senescent fibroblasts might be a source of the chronic inflammation that contributes cardiac collagen deposition and fibrosis formation after myocardial infarction.

In conclusion, the present study demonstrates that myocardial ischemia or hypoxia induced fibroblast senescence, increased cytokine expression and collagen deposition. These effects were associated with activation of p53 signaling pathway. Thus, inhibition of p53 activity could represent a novel therapeutic target for the reparative cardiac fibrosis and rupture after myocardial infarction.
